# Effect of Apolipoprotein E isoforms on the Abundance and Function of P-glycoprotein in Human Brain Microvascular Endothelial Cells

**DOI:** 10.1007/s11095-024-03731-0

**Published:** 2024-06-27

**Authors:** Ethan Kreutzer, Jennifer L. Short, Joseph A. Nicolazzo

**Affiliations:** 1 Drug Delivery, Disposition and Dynamics, Monash Institute of Pharmaceutical Sciences, Monash University, 381 Royal Parade, Parkville, Victoria 3052 Australia; 2 Monash Centre for Advanced mRNA Medicines Manufacturing and Workforce Training, Monash University, Clayton, Victoria 3800 Australia

**Keywords:** Alzheimer’s disease (AD), apolipoprotein E (apoE), blood-brain barrier (BBB), P-glycoprotein (P-gp)

## Abstract

**Background:**

Individuals with Alzheimer’s disease (AD) often require many medications; however, these medications are dosed using regimens recommended for individuals without AD. This is despite reduced abundance and function of P-glycoprotein (P-gp) at the blood-brain barrier (BBB) in AD, which can impact brain exposure of drugs. The fundamental mechanisms leading to reduced P-gp abundance in sporadic AD remain unknown; however, it is known that the apolipoprotein E (apoE) gene has the strongest genetic link to sporadic AD development, and apoE isoforms can differentially alter BBB function. The aim of this study was to assess if apoE affects P-gp abundance and function in an isoform-dependent manner using a human cerebral microvascular endothelial cell (hCMEC/D3) model.

**Methods:**

This study assessed the impact of apoE isoforms on P-gp abundance (by western blot) and function (by rhodamine 123 (R123) uptake) in hCMEC/D3 cells. Cells were exposed to recombinant apoE3 and apoE4 at 2 – 10 µg/mL over 24 – 72 hours. hCMEC/D3 cells were also exposed for 72 hours to astrocyte-conditioned media (ACM) from astrocytes expressing humanised apoE isoforms.

**Results:**

P-gp abundance in hCMEC/D3 cells was not altered by recombinant apoE4 relative to recombinant apoE3, nor did ACM containing human apoE isoforms alter P-gp abundance. R123 accumulation in hCMEC/D3 cells was also unchanged with recombinant apoE isoform treatments, suggesting no change to P-gp function, despite both abundance and function being altered by positive controls SR12813 (5 µM) and PSC 833 (5 µM), respectively.

**Conclusions:**

Different apoE isoforms have no direct influence on P-gp abundance or function within this model, and further *in vivo* studies would be required to address whether P-gp abundance or function are reduced in sporadic AD in an apoE isoform-specific manner.

## Introduction

Alzheimer’s disease (AD), a neurodegenerative condition resulting in cognitive decline, is a leading cause of death worldwide, which can be subcategorised into familial AD (FAD) and sporadic AD (SAD). FAD has a typical early onset, usually occurring before the age of 65, and accounts for approximately 1% of total AD cases, with well-defined genetic mutational causes [1]. SAD typically occurs later in life and its cause is less well defined. Age remains the greatest risk factor for SAD, alongside other factors including the gene for apolipoprotein E (apoE), which is the most established genetic risk factor for SAD development [2]. Individuals with AD often take five or more medications for both their AD symptoms and other comorbidities [3], yet these dosing regimens do not factor in possible AD-related changes to pharmacokinetics. AD may result in impacts on central nervous system (CNS) drug exposure given alterations to the blood-brain barrier (BBB) have been reported, including changes to key drug transport systems [4].

The BBB is the critical interface between the blood and the brain and plays an essential role in the regulation of molecular transport into and out of the brain. The BBB consists of endothelial cells and the tight junction proteins between these cells, restricting paracellular transport [5]. Several transport mechanisms also exist at the BBB, including efflux transporters, such as P-glycoprotein (P-gp), which have many drug substrates [6]. P-gp has received particular interest in AD due to being implicated in amyloid-β (Aβ) efflux [7]. Changes to P-gp at the BBB have already been documented in AD, including a reduction in P-gp abundance in FAD mouse models [8–10]. Similarly, studies with human AD brain samples have also shown a reduction in P-gp abundance [11, 12], whilst positron emission tomography scanning in humans with AD has suggested a reduction in P-gp function at the BBB [13]. Polymorphisms to this transporter have been investigated as a possible link to P-gp dysfunction in AD [14], and the reduced abundance of the protein transporter is suggested to play a role in AD pathology itself [15], given P-gp is reported to clear the neurotoxin Aβ from the brain [16]. However, many of these studies investigating P-gp abundance and function are often undertaken in FAD models, and the effect of apoE, the strongest genetic link to SAD development, on P-gp abundance and function remains under investigated.

ApoE is a 34 kDa glycoprotein which is associated with triglyceride and cholesterol-enriched lipoproteins, for the purposes of lipid and cholesterol trafficking to tissues. In humans, apoE has three different isoforms, apoE2, apoE3 and apoE4, which differ from each other via two single nucleotide polymorphisms [17]. Whilst the *APOE3 (E3)* allele is the most prevalent in the Caucasian population [18], possession of the *APOE4 (E4)* allele has been linked to SAD development, with possession of one allele copy increasing SAD risk four-fold and possession of two copies increasing that risk to greater than ten-fold, when compared to *E3* homozygotes [2]. Age of SAD onset is also decreased with possession of the *E4* allele [18]. The liver is the primary site for apoE synthesis and the brain is the second largest source [19], with astrocytes being the cell type responsible for producing a large proportion of the apoE found in the brain [20]. The peripheral and CNS apoE pools are kept separate by the BBB [21, 22]. Given its links to AD development and local synthesis within the CNS, there has been an increasing interest in the role of apoE in AD pathogenesis and its impact on the BBB.

There have been many studies demonstrating associations between possession of the *E4* allele and AD hallmark pathology including Aβ accumulation [23–26] and increased tau levels [27]; however, apoE4 is also involved in BBB dysfunction. This has been reported as a reduction in tight junction proteins in *E4* knock-in mice relative to mice producing apoE3 [28], although this association was not observed in post-mortem human samples [29]. Increased BBB permeability, as determined by magnetic resonance imaging, has been observed in humans carrying the *E4* allele and reported to predict cognitive decline independent of AD pathology changes such as Aβ and tau levels [30]. A reduction in overall pericyte coverage of the BBB in mice expressing *E4* compared to *E3* mice, in addition to accelerated neuron loss [31], is also suggestive of the detrimental effects of the apoE4 isoform on cell types responsible for regulating the BBB. Acceleration in reduced pericyte coverage associated with apoE4 has also been observed in the human cortex [32]. A multi-omics analysis study reported progressive BBB breakdown which preceded synaptic changes in *E4* knock-in mice relative to *E3* knock-in mice [33]. Changes to the BBB paracellular route due to apoE4 isoform status may in turn alter the functionality of the BBB, which becomes particularly pertinent to the access of medications into the CNS.

One study assessing apoE status on CNS drug access found reduced brain uptake of the passively-diffusing molecule diazepam in mice expressing *E4* compared to *E3* and *E2* [34]. This was attributed to a reduced brain microvascular surface area and subsequent reduction in cerebral vascularisation in the *E4 *mice [34]; however, this study did not assess the impact of apoE isoforms on key BBB transporters such as P-gp. A reduction in P-gp abundance and function has been observed in a transgenic mouse model of FAD that had been crossed to express *E4* [35]. However, the mechanism for this reduction remains unclear and it is not known whether the interplay between apoE and AD pathology led to these observations or whether it was a direct apoE effect. Nevertheless, this suggests that there may be an interplay between apoE isoform status and P-gp.

The aim of this study, therefore, was to investigate the impact of different apoE isoforms on P-gp abundance and function in human brain microvascular endothelial cells. Whilst previous studies have offered insight into BBB changes in AD, these studies have primarily been centred around FAD models. Given the link between apoE isoform status and SAD development, the results of this study would not only provide insight into potential molecular changes for the greater proportion of individuals with AD, but also potentially lead to a better understanding of how apoE genotype impacts drug transporters and in turn CNS drug exposure.

## Materials and Methods

### General Materials & Reagents

Dulbecco’s phosphate-buffered saline (D-PBS) (Cat.#D8537), dimethyl sulfoxide (DMSO) (Cat.#41640), HEPES (Cat.#H4034), Tris base (Cat.#T1503), glycine (Cat.#G8898), sodium chloride (Cat.#S3014), sodium dodecyl sulfate (SDS) (Cat.#L3771), TWEEN^®^ 20 (Cat.#P2287), Triton™ X-100 (Cat.#T8787), cOmplete™ Mini Protease Inhibitor Cocktail (Cat.#04693124001), SR12813 (Cat.#S4194), rhodamine 123 (R123) (Cat.#R8004), thiazolyl blue tetrazolium bromide (MTT) (Cat.#M5655), Trypan blue solution (Cat.#T8154), penicillin-streptomycin (Cat.#P4458), Corning^®^: T25 flasks (Cat.#CLS430639), 6-well plates (Cat.#CLS3516), 48-well plates (Cat.#CLS3548), 96-well plates (Cat.#CLS3596), 50 mL centrifuge tubes (Cat.#CLS430829) and 15 mL centrifuge tubes (Cat.#CLS430791) were purchased from Sigma-Aldrich (St. Louis, MO). PSC 833 (Cat.#20391) was purchased from Cayman Chemical (Ann Arbor, MI). Ultrapure™ DNase/RNase-free distilled water (Cat.# 10977015), Hank’s balanced salt solution (HBSS) (Cat.#14025134), Pierce™ IP lysis buffer (Cat.#87788), Pierce™ BCA protein assay kit (Cat.#23225), Invitrogen™ Apolipoprotein E human ELISA kit (Cat.#EHAPOE), Geneticin™ selective antibiotic (Cat.#10131035), sodium pyruvate (Cat.#11360070), poly-d-lysine (Cat.#A3890401), trypsin-EDTA (0.25%) (Cat.#25200072), and Gibco™ Advanced DMEM (Cat.#12491015) were purchased from Thermo Fisher Scientific (Waltham, MA). Endothelial basal medium-2 (EBM2) media (Cat.#190860) was supplemented with a growth factor kit (Cat.#CC-4176) purchased from Lonza (Walkersville, MD). Foetal bovine serum (FBS) (Cat.#SFBS-AU) was purchased from Bovogen Biologicals (Melbourne, Victoria, Australia), HyClone™ bovine serum albumin (Cat.#SH30574.02) was purchased from Cytiva Life Sciences (Marlborough, MA) and rat-tail collagen type I (Cat.#354236) was purchased from Corning (Corning, NY). 4-15% Mini-PROTEAN^®^ TGX™ Precast Protein Gels (Cat.#4561084), extra thick blot filter paper (Cat.#1703960), 0.45 µm nitrocellulose membrane (Cat.#1620115) and Precision Plus Protein™ dual Xtra prestained protein standards (Cat.#1610377) were purchased from Bio-Rad (Hercules, CA). Intercept^®^ (PBS) Blocking Buffer (Cat.#927-70003), IRDye^®^ 800CW Goat Anti-Mouse IgG (Cat.#926-32210) and IRDye^®^ 680LT Donkey Anti-Rabbit IgG (Cat.#926-68023) were purchased from LI-COR Biosciences (Lincoln, NE). Primary C219 monoclonal antibody for P-gp (Cat# 903701) was purchased from BioLegend (San Diego, CA) and primary monoclonal antibody for β-actin (Cat.#ab179467), recombinant human apolipoprotein E3 (Cat.#ab123764) and recombinant human apolipoprotein E4 (Cat.#ab50243) were purchased from Abcam (Cambridge, UK). Amicon^®^ Ultra-15 Centrifugal Filter Devices (Cat.#UFC901096) were purchased from Merck Millipore (Burlington, MA). Microcentrifuge tubes (Cat.#P4010) were purchased from Rowe Scientific (Wangara, Western Australia, Australia) and T75 flasks (Cat.#658175) were purchased from Interpath Services (Somerton, Victoria, Australia). Milli-Q (MQ) water was obtained from a Millipore system (Billerica, MA).

### Culture of Human Cerebral Microvascular Endothelial Cell (hCMEC/D3) Line

The hCMEC/D3 cell line was kindly supplied by Dr Pierre-Olivier Couraud (Inserm, Paris, France). These cells were kept between passages 29 and 35 to limit cellular drift and phenotypic changes [36]. hCMEC/D3 cells were also purchased from Merck Millipore (Cat.#SCC066) and similarly kept under 10 passages to prevent expressional and functional changes. hCMEC/D3 cells were cryopreserved before resuscitation, seeding and splitting for studies, as previously described by our laboratory [37]. Briefly, cell vials were removed from liquid nitrogen storage and the cells were seeded in T75 flasks coated with 0.1 mg/mL collagen type I and containing 14 mL of growth media referred to as EBM2+, consisting of EBM2 supplemented with: 0.01% (v/v) ascorbic acid, 0.01% (v/v) gentamicin/amphotericin, 0.01% (v/v) hydrocortisone, 0.025% (v/v) epidermal growth factor, 0.025% (v/v) insulin-like growth factor, 0.025% (v/v) vascular endothelial growth factor, 0.1% (v/v) b-splice variant fibroblast growth factor, 10 mM HEPES, 1% (v/v) penicillin/streptomycin and 2.5% (v/v) FBS. Flasks were incubated at 37ºC with 5% CO_2_. After two hours, fresh EBM2+ was replaced in flasks and cells were allowed to grow to at least 80% confluency before splitting, replacing with fresh growth media every 48 hours. Cells were washed with D-PBS twice prior to splitting via exposure to trypsin/EDTA solution. Trypsin activity was stopped with 2.5% (v/v) FBS in D-PBS and the cell suspension was centrifuged at 650 RCF for 5 minutes at 25ºC. Counting of cells was undertaken using a haemocytometer with Trypan blue staining. Cells were then seeded at a density of 20,000 cells/cm^2^ for studies with 6-well plates being used for protein abundance studies, 48-well plates for functional studies and 96-well plates for viability studies. In these studies, hCMEC/D3 cells were either treated with recombinant apoE protein isoforms or conditioned media collected from cultured immortalised astrocytes producing humanised apoE isoforms to assess their impact on P-gp abundance and function.

### Culture of Immortalised Astrocytes Derived from Human APOE Knock-in Mice

Immortalised astrocytes originally produced and characterised by Morikawa *et al*. [38] were kindly provided by A/Prof. Lance Johnson (University of Kentucky, KY). These immortalised astrocytes are respectively homozygous for either human *E2, E3 or E4* and secrete human apoE at detectable levels [38]. All cells used in studies were kept between passages 16 and 20 in growth media referred to as Adv. DMEM+, consisting of Advanced DMEM supplemented with 10% (v/v) FBS, 1% (v/v) sodium pyruvate and 0.4% (v/v) Geneticin™ selective antibiotic. Cells were cryopreserved in freezing media consisting of Adv. DMEM+ and 10% (v/v) DMSO, and stored in liquid nitrogen prior to thawing and seeding for studies. Prior to cell resuscitation, T25 flasks were coated with 500 µL of poly-d-lysine and incubated for two hours at 37ºC. After two hours, poly-d-lysine was removed, flasks were washed once with D-PBS and left to dry. Once dry, cells were removed from cryopreservation, transferred to a 15 mL tube containing 10 mL of Adv. DMEM+ and centrifuged at 310 RCF for 5 minutes at 25ºC. Media was then carefully removed, and the cell pellet was resuspended in 5 mL of Adv. DMEM+ before seeding the T25 flask with this 5 mL cell suspension. Cells were grown in T25 flasks until at least 80% confluency, replacing growth media every 48 hours before splitting into T75 flasks. Briefly, 1 mL of trypsin/EDTA solution was added to T25 flasks and allowed to incubate at 37ºC with 5% CO_2_ for 2 – 3 minutes. Detachment of cells was confirmed under the microscope and 10 mL of Adv. DMEM+ was added to arrest trypsin activity. Contents of the flask were transferred to a 15 mL tube and centrifuged at 310 RCF for 5 minutes at 25ºC. The media supernatant was then aspirated, and cells were resuspended in 5 mL of growth media. Astrocytes were seeded at either a 1:10 or 1:15 ratio (cell suspension:growth media) dependent on the desired growth rate, with a final volume of 12 mL of growth media in T75 flasks. Astrocytes were allowed to grow until approximately 100% confluency and at this stage, the conditioned growth media from flasks was collected.

### Collection and Concentration of Astrocyte-Conditioned Media (ACM)

Astrocyte-conditioned media (ACM) containing secreted human apoE isoforms was collected from immortalised astrocyte lines at the point of approximately 100% confluency. ACM from *E2*, *E3* and *E4* astrocytes was collected after both 24 and 48 hours of growth of immortalised astrocytes. Briefly, conditioned media was removed from flasks at either 24 or 48 hours, transferred to a 15 mL tube and centrifuged at 3245 RCF for 10 minutes at 4ºC to remove any debris. ACM was then transferred to a new 15 mL tube and stored at -80ºC before use in treatments in either unconcentrated or concentrated volumes.

For concentration of ACM, the relevant conditioned media was thawed from -80ºC and transferred to centrifugal filter devices with a nominal molecular weight limit of 10 kDa chosen to capture the 34 kDa apolipoprotein. The devices were centrifuged at 4000 RCF for 20 minutes at 4ºC based on the manufacturer’s recommendations. Concentrated ACM was then collected from the filter device sample reservoir using a pipette, and stored at -80ºC before use in treatments or analysis.

### Cell Treatments

For all protein abundance analyses, hCMEC/D3 cells were grown in 6-well plates. Lyophilised recombinant apoE3 (rE3) and recombinant apoE4 (rE4) were reconstituted in sterile distilled H_2_O to a concentration of 1 mg/mL before storage at -20ºC. hCMEC/D3 cells, which were seeded at 20,000 cells/cm^2^, were exposed to rE3 or rE4 at 2 µg/mL for 24 and 48 hours or rE3 or rE4 at 2 µg/mL and 10 µg/mL for 72 hours. A concentration of 2 µg/mL was chosen as it reflects the level of apoE that has been reported to be secreted from the immortalised astrocyte lines [38], as well as in the cerebrospinal fluid (CSF) of targeted-replacement *APOE* mice [39]. A concentration of 10 µg/mL was used given that human CSF levels of apoE have been reported to be an average of approximately 9 µg/mL in one study [40], and ranging from 5.7 to 8.9 µg/mL in another study [41]. As a positive regulator of P-gp, hCMEC/D3 cells were exposed to 5 µM of SR12813 for 48 or 72 hours [42, 43].

For ACM treatments, unconcentrated ACM was added to hCMEC/D3 growth media (EBM2+) in a 1:1 or 1:2 ratio (ACM:EBM2+) and this solution was used to treat hCMEC/D3 cells for a 48 hour period. In separate studies, concentrated ACM was used to treat the hCMEC/D3 cells by mimicking the 1:1 unconcentrated treatment ratio. For example, if 5 mL of unconcentrated ACM was required to make the treatment solution and 10 mL of ACM, once concentrated, yielded 1 mL of concentrate, then 500 µL of the concentrated ACM would be used in treatments to reflect the required 5 mL, assuming negligible loss of apoE through the filter device. Cells were treated with concentrated ACM for a 72 hour period. ApoE content was also determined by enzyme-linked immunosorbent assay (ELISA) and the concentration of ACM adjusted in separate studies to normalise for apoE content across isoforms.

#### Quantification of apoE by ELISA

To quantify the amount of apoE secreted by the immortalised astrocyte lines, a sandwich ELISA was used. A commercial human apoE ELISA kit was utilised with standard operating procedures followed as per the manufacturer’s protocols. Briefly, recombinant human apoE standards and ACM samples were diluted as required and 100 µL of both standards and samples were added in duplicate to human apoE antibody-coated wells in a 96-well plate. Wells were covered and incubated overnight with gentle agitation at 4ºC, after which, solutions were discarded and the plate washed four times with wash buffer. Diluted biotin-conjugated secondary antibody was added to wells and the plate incubated for an hour at room temperature with gentle agitation. Solutions were then removed and wells were washed a further four times. Streptavidin-horseradish peroxidase was added and the plate was allowed to incubate for a further 45 minutes at room temperature with gentle agitation. Solutions were again removed and a final four washes undertaken. 3,3′,5,5′-Tetramethylbenzidine substrate was added to each well and the plate incubated for a final 30 minutes in the dark at room temperature with gentle agitation, after which the stop solution was added, and absorbance read at 450 nm using an Enspire absorbance spectrophotometere (PerkinElmer, Waltham, MA).

#### Cell Viability Assessment

hCMEC/D3 cells were seeded into 96-well plates at 20,000 cells/cm^2^ for the purposes of assessing cell viability post treatment with recombinant apoE or ACM. Treatment periods were 48 or 72 hours, after which treatments were removed, cells were rinsed once with warm D-PBS and 150 µL of 0.45 mg/mL MTT (thiazolyl blue tetrazolium bromide) in FBS-free EBM2 was added to wells. Cells were then incubated for 4 hours at 37ºC with 5% CO_2_ before excess MTT solution was removed and 150 µL of sterile DMSO was added to each well. Cells were incubated for a further 30 minutes at 37ºC with 5% CO_2_ before the absorbance was measured at 540 nm using an Enspire absorbance spectrophotometer. Viability was calculated as a percentage of the absorbance in treated cells relative to the absorbance of vehicle control treated cells after subtracting background absorbance.

#### Western Blotting Analysis of P-gp Abundance

Following treatment of hCMEC/D3 cells with recombinant apoE or ACM, cells were removed from incubation and lysed in 200 µL of Pierce™ IP Lysis Buffer containing cOmplete™ Mini Protease Inhibitor Cocktail for 20 minutes at 4ºC with gentle shaking. Cell lysates were collected and centrifuged at 14,000 RCF for 10 minutes at 4ºC, and lysate samples stored at -80ºC prior to use. Total protein content was determined via a bicinchoninic acid (BCA) assay which had been validated for precision (coefficient of variation < 5%) and accuracy (100% ± 10%) using the protocols specified in the Pierce™ BCA protein assay kit. Lysate volumes containing 10 µg of protein, as determined by BCA assay, were combined with 6X Laemmli buffer in a 5:1 ratio, briefly vortexed and incubated for 20 minutes at 37ºC. Samples were then again vortexed and centrifuged at 14,000 RCF for 3 minutes at room temperature. Samples (10 µg) were loaded onto a 4-15% Mini-PROTEAN^®^ TGX™ Precast Protein Gel, alongside Precision Plus Protein™ Dual Xtra Prestained Protein Standards, which was then assembled into the Bio-Rad Mini-PROTEAN^®^ Tetra Cell. The gel was submerged in cold running buffer (25 mM Tris base, 192 mM glycine, 0.1% (w/v) SDS in MQ water) and the Tetra Cell apparatus surrounded with ice. Electrophoresis was run at 60V for 30 minutes followed by 150V for approximately one hour. The gel was subsequently removed from the apparatus and incubated for 20 minutes in cold transfer buffer (25 mM Tris base, 192 mM glycine, 20% (v/v) methanol, 0.02% (w/v) SDS in MQ water), as were extra thick blotting paper and a 0.45 µm pore-size nitrocellulose membrane. Protein was transferred to the membrane using a Bio-Rad Trans-Blot Turbo transfer system (Hercules, CA) at 25V and 1.0A for 40 minutes. After transfer, the membrane was washed with Tris-buffered saline (50 mM Tris base, 154 mM NaCl in MQ water, pH 7.6) containing 0.05% (v/v) TWEEN^®^ 20 (TBS-T), and then blocked with Intercept^®^ (PBS) Blocking Buffer for 90 minutes at room temperature with gentle agitation.

After blocking, the membrane was rinsed with TBS-T and co-incubated with primary antibodies for P-gp (1:1000) and β-actin (1:500,000) overnight at 4ºC with gentle shaking. Primary antibody solutions were subsequently decanted, and the membrane was washed for 5 minutes four times with 20 mL of TBS-T under constant, gentle agitation. Following washing, the membrane was co-incubated with secondary antibodies IRDye^®^ 800CW Goat Anti-Mouse IgG (1:7500) and IRDye^®^ 680LT Donkey Anti-Rabbit IgG (1:15000) for two hours at room temperature with gentle shaking in a lightproof box. Washing steps were repeated after secondary incubation and the membrane was then scanned for protein band detection using an Amersham Typhoon 5 biomolecular imager (Cytiva, Marlborough, MA). ImageJ software (National Institutes of Health, Bethesda, MD) was used to perform densitometric analysis of P-gp protein bands which were then normalised to the housekeeping protein β-actin.

#### Assessment of P-gp Function

Accumulation of R123, a fluorescent P-gp substrate, in hCMEC/D3 cells was used to assess P-gp function. Cells were seeded at 20,000 cells/cm^2^ in 48-well plates and treated for a 48 hour period with recombinant apoE isoforms, after which treatments were removed and cells washed twice with warm D-PBS. Cells were then incubated with transport buffer (10 mM HEPES in HBSS, pH 7.4) for 15 minutes at 37ºC with 5% CO_2_, after which transport buffer was replaced with pre-warmed transport buffer containing R123 (5 µM) and cells were incubated for 60 minutes at 37ºC with 5% CO_2_ under constant, gentle agitation. After 60 minutes, cells were removed from incubation and washed three times with ice-cold transport buffer and then lysed with 1% (v/v) Triton X-100 in MQ water for 20 minutes at 4ºC. Lysate samples were transferred to a 96-well plate alongside R123 standards of known concentrations, with fluorescence then measured at an excitation wavelength of 511 nm and an emission wavelength of 534 nm. Lysate samples were subsequently assessed for total protein content via the BCA assay, as previously described. The amount of R123 accumulation was then normalised to total protein content and compared between groups. PSC 833, a known P-gp inhibitor [44], was included as a positive control. hCMEC/D3 cells were incubated with 5 µM of PSC 833 in transport buffer for 15 minutes at 37ºC with 5% CO_2_ before removal of solutions and a subsequent 60 minute co-incubation with R123 (5 µM) and PSC 833 (5 µM) at 37ºC with 5% CO_2_. After 60 minutes, cells were lysed and analysed for fluorescence and total protein content as described above.

#### Statistical Analysis

Data generated from studies were analysed with GraphPad^®^ prism version 9.5.1 (GraphPad Software, Boston, MA). Experimental groups were compared via an unpaired t-test or one-way analysis of variance (ANOVA) dependent on the number of groups for comparison and study design. In the case of a one-way ANOVA, this was followed by a post-hoc analysis, such as a Dunnett’s test or Tukey’s test. Statistical significance was defined as a p-value less than 0.05.

## Results

### Recombinant apoE Isoform Treatments Reduce hCMEC/D3 Cell Viability

**Fig. 1:**
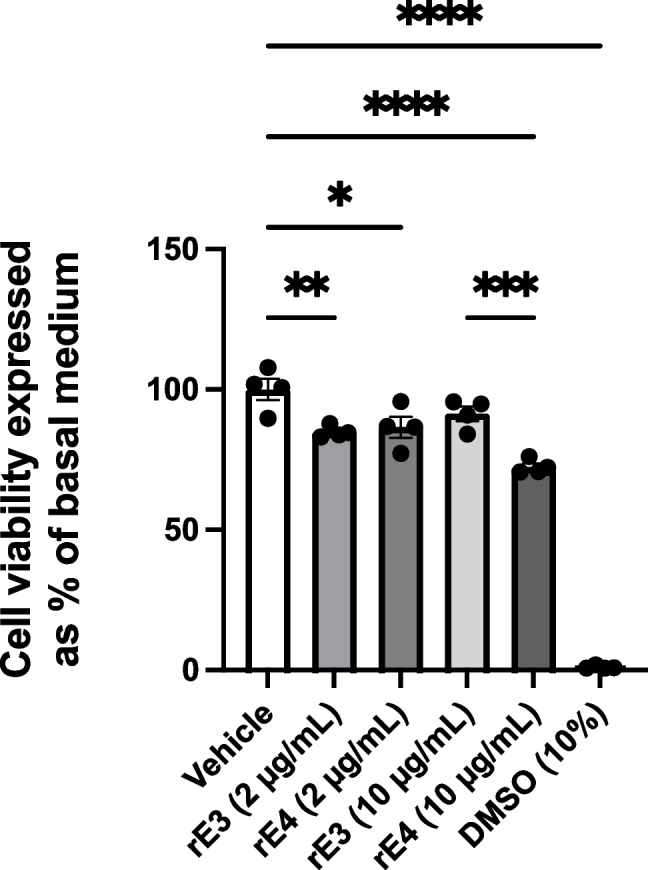
MTT cell viability assay of hCMEC/D3 cells after a 72 hour treatment with rE3 and rE4 at either 2 µg/mL or 10 µg/mL concentration. DMSO 10% (v/v) was used as a positive control for acute cell toxicity. Data presented as mean ± SEM (n=4) expressed as % of basal growth medium (i.e., EBM2+) when assessed by one-way ANOVA followed by a post-hoc Tukey’s test, *p<0.05, **p<0.01, ***p<0.001, ****p<0.0001.

To determine the impact of apoE recombinant isoform treatments on hCMEC/D3 cell viability, an MTT assay was conducted. Given the links between apoE4 and AD and the prevalence of the *E3* allele, rE3 and rE4 were chosen as the initial isoforms of interest. hCMEC/D3 cells were treated with rE3 and rE4 at both 2 µg/mL and 10 µg/mL for 72 hours. All the treatments, except the rE3 10 µg/mL, reduced hCMEC/D3 cell viability, by approximately 14 – 28% (Fig. 1). However, the most important comparisons were between rE3 and rE4 at both concentrations. A concentration of 2 µg/mL of either recombinant isoform reduced viability by approximately 14 – 15%; however, there was no significant difference observed between isoforms at this concentration. At the 10 µg/mL concentration, rE4 led to a statistically significant reduction in viability (by 28%), with rE4 resulting in an approximate 19% reduction compared to rE3 at this concentration. At a 10 µg/mL concentration, rE3 treatment led to a reduction by approximately 9% compared to the vehicle control, although this did not reach statistical significance. These findings were taken into consideration for subsequent studies; however, as visual inspections of cultures confirmed cells treated with recombinant proteins were still able to proliferate, functional and abundance studies still proceeded.

### Recombinant apoE3 and apoE4 Isoforms do not have an Effect on P-gp Abundance and Function in hCMEC/D3 Cells

**Fig. 2:**
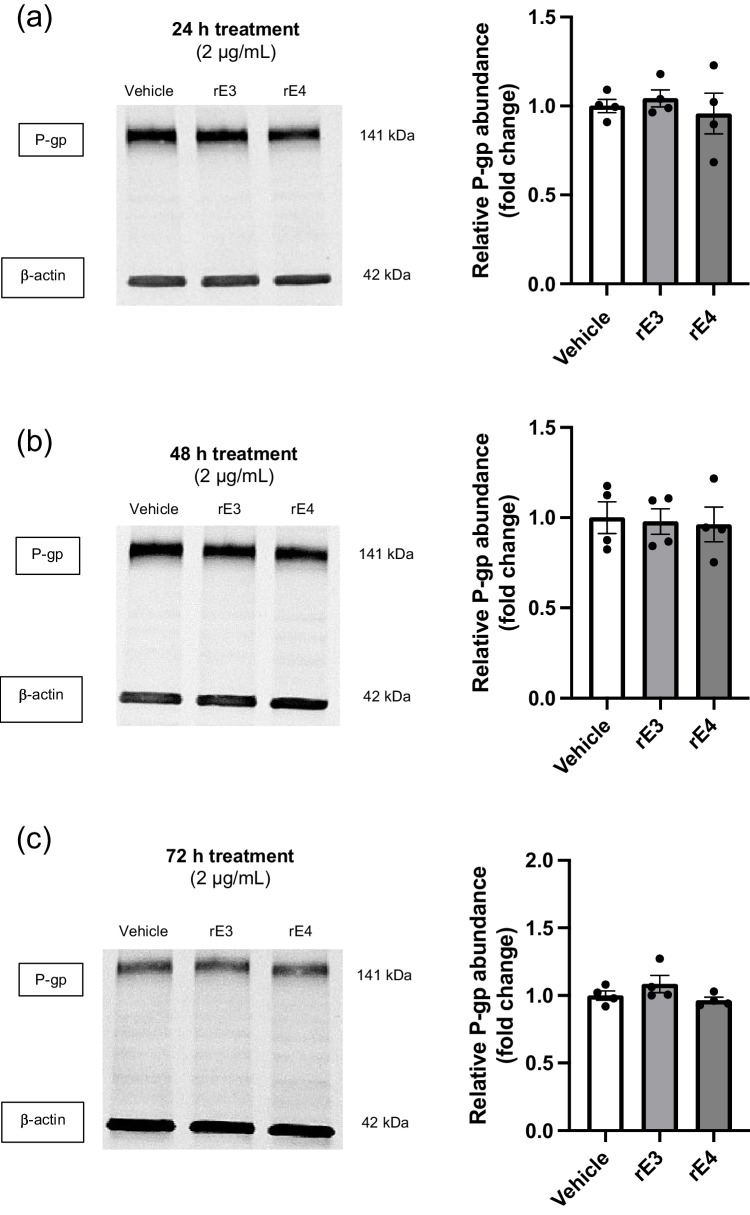

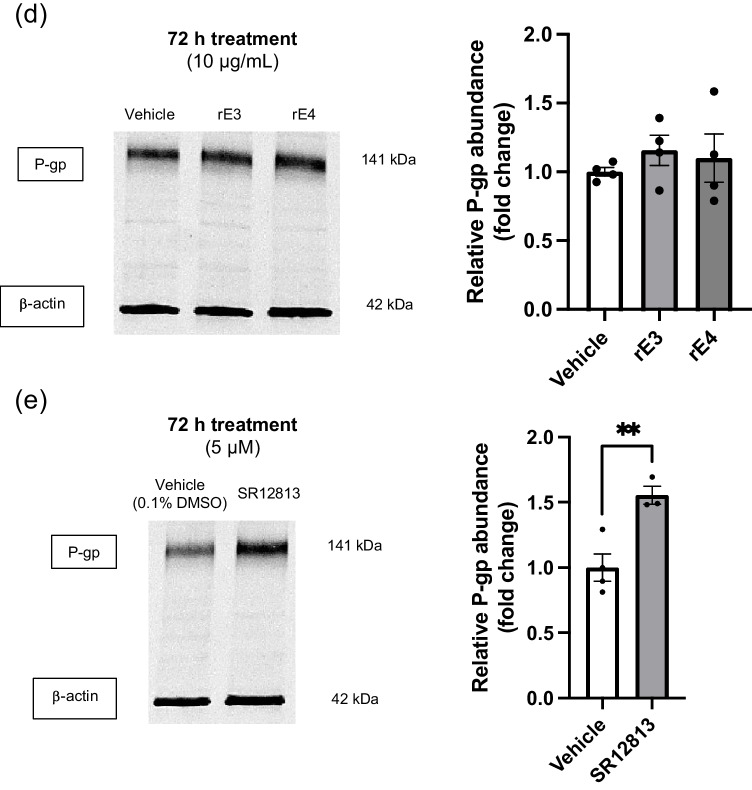
Recombinant apoE3 (rE3) and apoE4 (rE4) do not directly modulate abundance of P-gp in hCMEC/D3 cells. Representative western blots of P-gp and β-actin protein abundance levels grouped from a single gel after (**a**) 24 hour and (**b**) 48 hour exposure with 2 µg/mL of rE3 or rE4, **(c**) 72 hours at 2 µg/mL of rE3 or rE4, and (**d**) 72 hours at 10 µg/mL of rE3 or rE4. (**e**) A 72 hour treatment with 5 µM SR12813 was used a positive control for changes in P-gp abundance. Graphical representation of abundance as assessed by densitometry is presented as mean ± SEM (n=3-4) expressed as a fold change of vehicle control when assessed by (**a**-**d**) one-way ANOVA, or (**e**) an unpaired t-test, **p<0.01.

To assess whether the apoE isoforms had a direct isoform-dependent effect on P-gp abundance, rE3 and rE4 were reconstituted and used to treat hCMEC/D3 cells over 24 and 48 hours. Analysis of P-gp abundance post western blotting indicated no significant changes at any of the tested timepoints or concentrations when compared to vehicle control (Fig. 2a-d). There were also no isoform-dependent differences observed across all timepoints, suggesting that total P-gp abundance is not directly altered by apoE in an isoform-dependent manner. SR12813, which is known to upregulate P-gp, was used to treat hCMEC/D3 cells at a concentration of 5 µM and demonstrated that protein abundance of P-gp can be modified in this cellular model (Fig. 2e).

### ApoE Isoforms do not alter P-gp Efflux Function

**Fig. 3:**
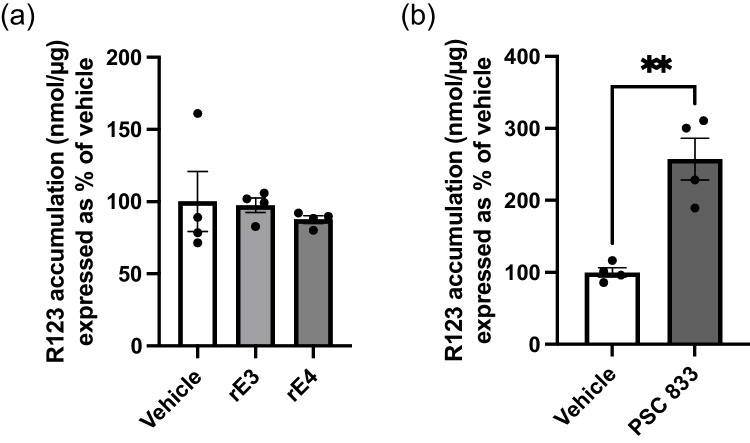
Recombinant apoE3 (rE3) and recombinant apoE4 (rE4) do not affect P-gp function as assessed via rhodamine 123 (R123) accumulation after 60 minutes. Treatment with (**a**) rE3 or rE4 (2 µg/mL) for 48 hours prior to functional assay and (**b**) PSC 833 (5 µM) which was used as a positive control for inhibition of P-gp. Data presented as mean ± SEM (n=4) expressed as % R123 accumulation of vehicle control when assessed by (**a**) one-way ANOVA and (**b**) unpaired t-test, **p<0.01.

As P-gp is a membrane-bound efflux transporter, functional studies were undertaken to determine whether apoE isoforms may have an impact on P-gp efflux activity, even if protein abundance was unaltered. Function was determined using R123, a known P-gp fluorescent substrate, with accumulation of R123 serving as a surrogate for efflux activity. PSC 833 was included as a positive control for inhibition of P-gp. hCMEC/D3 cells were treated with either rE3 or rE4 for 48 hours at 2 µg/mL. Accumulation of R123 did not differ after the 60 minute incubation when compared to vehicle control or between isoform groups (Fig. 3a). A significant increase in R123 accumulation was observed with 5 µM PSC 833 treatment (Fig. 3b), confirming that P-gp functionality could be modified. These results indicate that there is no difference in P-gp function following treatment with different apoE isoforms, in line with a lack of effect of apoE on P-gp abundance.

### Impact of Conditioned Media from *APOE* Astrocytes on hCMEC/D3 P-gp Abundance

**Fig. 4:**
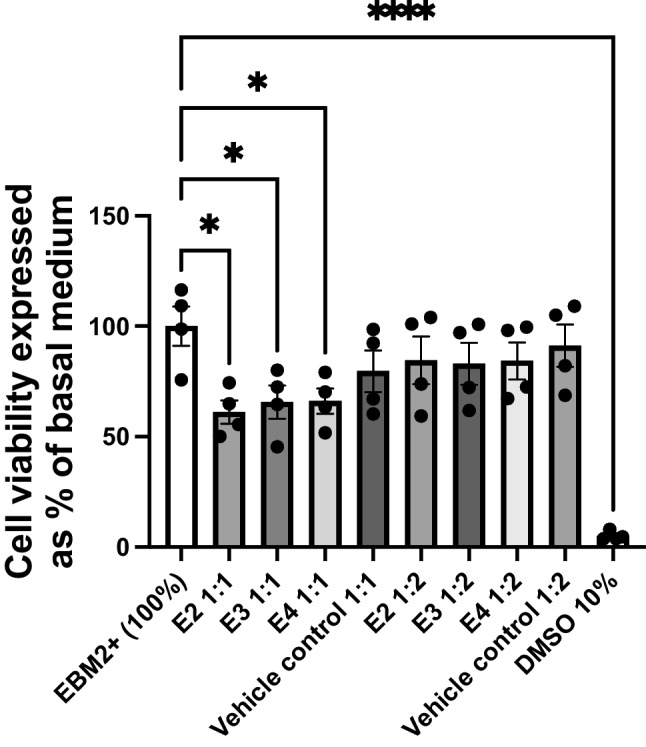
MTT cell viability assay of hCMEC/D3 cells after a 48 hour treatment with 1:1 and 1:2 ACM containing apoE2, apoE3 or apoE4, where X:Y ratio refers to X = ACM (in Adv. DMEM+) and Y = hCMEC/D3 growth media (EBM2+). Normal hCMEC/D3 growth media, EBM2+ (100%), was included as a baseline control. Vehicle controls of media mixes of Adv. DMEM+ and EBM2+ were also included. DMSO 10% (v/v) was used as a positive control for acute cell toxicity. Data presented as mean ± SEM (n=4) expressed as % of basal medium (i.e., EBM2+), when assessed by one-way ANOVA followed by a post-hoc Dunnett’s test, *p<0.05, ****p<0.0001.

Prior to assessing the impact of media from *E2*, *E3* and *E4* astrocytes on P-gp abundance in hCMEC/D3 cells, it was important to assess whether altering the normal growth media of hCMEC/D3 cells (by combining it with ACM in a 1:1 or 1:2 ratio) affected the viability of hCMEC/D3 cells. Treatment with a 1:2 ratio (growth media:ACM) did not produce significant cell toxicity, although all 1:1 treatment ratios exhibited a loss in viability, irrespective of apoE isoform, when compared to 100% growth media (Fig. 4). Further, the vehicle control 1:1 treatment group (a media mix of EBM2+ and Adv. DMEM+ in a 1:1 ratio) displayed no significant loss in viability compared to the 100% hCMEC/D3 growth media control, suggesting that conditioning of the media by astrocytes itself may be contributing to the reductions in cell viability observed.

**Fig. 5:**
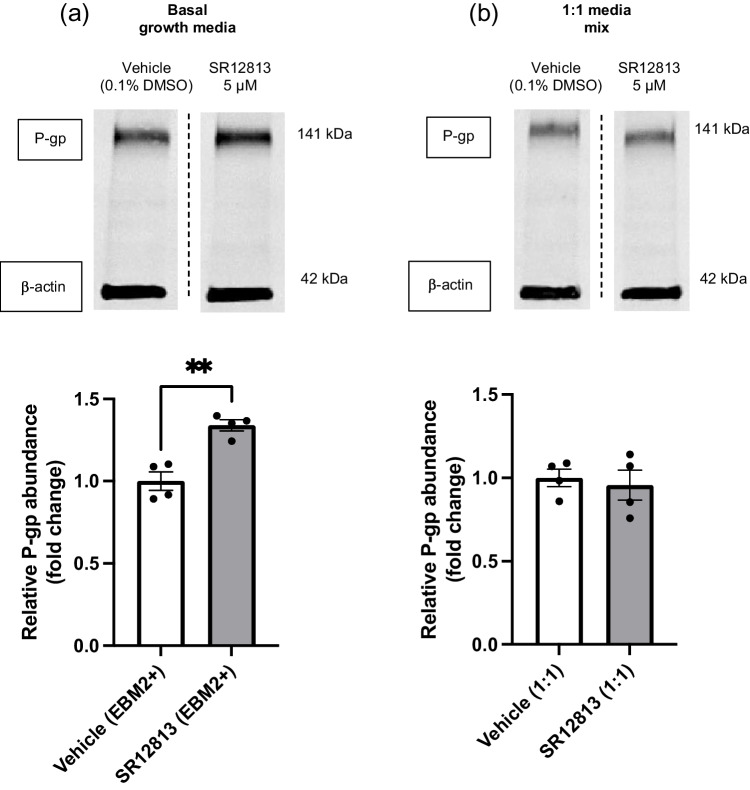
SR12813 at 5 µM increases P-gp abundance in hCMEC/D3 cells when in basal growth media (EBM2+) but not in 1:1 media mix environment (Adv DMEM+ : EBM2+). Representative western blots of P-gp and β-actin protein abundance levels when treated in (**a**) basal growth media or (**b**) 1:1 media mix for 48 hours. Protein bands grouped from a single gel (--- dividing lines indicative of reordered lanes for presentation only). Graphical representation of abundance as assessed by densitometry is presented as mean ± SEM (n=4) expressed as a fold change of vehicle control (0.1% (v/v) DMSO) when assessed by unpaired t-test, **p<0.01.

Despite the differences in cell viability observed with ACM treatments via the MTT assay, there were no differences between viability of hCMEC/D3 cells treated with the different apoE isoforms at a 1:1 ratio. Therefore, as inter-isoform differences in P-gp abundance were of interest, the cell culture system was first assessed for its ability to regulate and modify P-gp abundance within the 1:1 media ratio environment. SR12813 was used to treat hCMEC/D3 cells at a concentration of 5 µM over 48 hours in the vehicle control 1:1 media system as well as in normal hCMEC/D3 growth media (EBM2+) to ensure that P-gp is still able to be regulated when the basal media is modified. A significant increase in P-gp abundance by SR12813 was observed with the use of normal growth media (Fig. 5a). However, no such increase was observed when treating the cells in the 1:1 media mix (Fig. 5b), suggesting that this 1:1 media system is removing the ability of SR12813 to drive P-gp upregulation in hCMEC/D3 cells. Further, this suggests that the 1:1 media matrix employing unconcentrated ACM is unsuitable for the assessment of P-gp abundance, and an alternative system was therefore utilised.

### Immortalised Astrocytes Derived from Human *APOE* Knock-in Mice Produce apoE at Variable Quantities

**Fig. 6:**
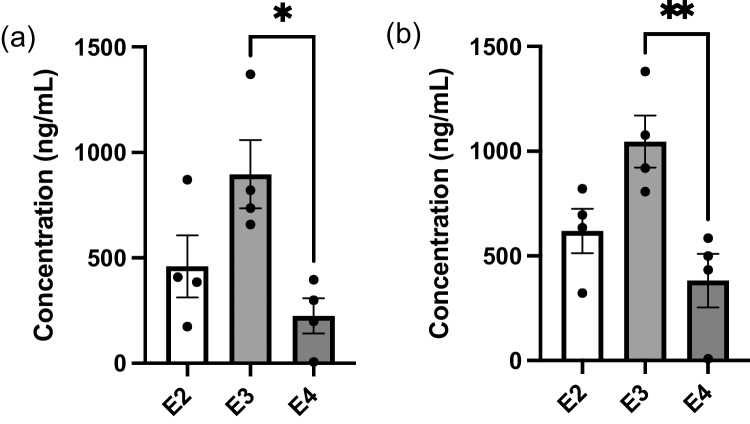
ApoE levels secreted from immortalised astrocytes derived from targeted-replacement *APOE* mice are highly variable. ApoE concentration as assessed via ELISA from immortalised astrocyte cultures after (**a**) 24 hours and (**b**) 48 hours of media conditioning. Data presented as mean ± SEM (n=4) expressed as concentration (ng/mL) when assessed by one-way ANOVA followed by a post-hoc Tukey’s test, *p<0.05, **p<0.01.

To assess the homogeneity of humanised apoE secretion from immortalised astrocytes, ACM was collected from astrocyte cultures at different passages at the point of confluency, after 24 and 48 hours of conditioning by cells. Collected ACM was then analysed via sandwich ELISA targeted against human apoE. Blank Adv. DMEM+ and sample media from hCMEC/D3 cell culture were included as controls to confirm the absence of external apoE or other confounding sources that may influence the results. No apoE was found in either the blank media or from growth media collected from hCMEC/D3 culture (data not shown). ELISA results from the collected ACM samples demonstrated an apoE production pattern of apoE3>apoE2>apoE4 at both 24 hours (Fig. 6a) and 48 hours (Fig. 6b) of conditioning by astrocyte cultures. While the levels of apoE produced from astrocytes were highly variable, the ACM containing apoE3 reached statistical significance over the ACM containing apoE4. Whilst conditioning time and passage number may account for some of the variability observed in apoE content, it appears that apoE is produced by immortalised astrocytes at vastly different quantities both between isoform types and within isoform groups themselves, making it challenging to consistently isolate and collect secreted apoE for treatment purposes. However, concentrations of apoE determined by ELISA were subsequently used to inform the volumes of ACM used for concentration-corrected treatments.

### Concentrated ACM has no apoE Isoform-Dependent Effect on Abundance or Function of P-gp in hCMEC/D3 Cells

**Fig. 7:**
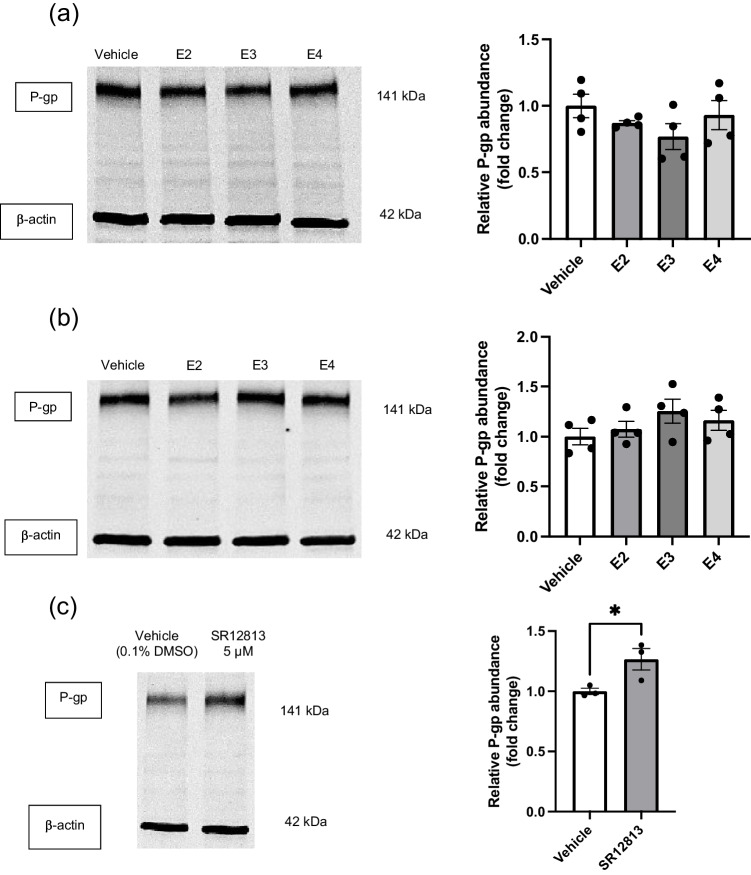
P-gp abundance is unchanged by apoE isoforms produced by astrocytes in ACM treatments. Representative western blots of P-gp and β-actin protein abundance levels grouped from a single gel after a 72 hour treatment with (**a**) ACM mimicking the 1:1 media mix content of apoE and (**b**) concentration-corrected ACM by apoE content. (**c**) 5 µM SR12813 was used as a positive control for changes in P-gp abundance within the concentrated media system. Graphical representation of abundance as assessed by densitometry presented as mean ± SEM (n=3-4) expressed as a fold change of vehicle control when assessed by (**a**) & (**b**) one-way ANOVA, or (**c**) an unpaired t-test, *p<0.05.

To uncover if an alternative system using ACM was viable in exploring changes to P-gp abundance, collected ACM was concentrated, allowing a smaller total volume to be used. Firstly, ACM containing apoE2, apoE3 or apoE4 was collected and used to treat hCMEC/D3 cells for 72 hours, mimicking the 1:1 unconcentrated ratio treatment described above. SR12813 at 5 µM was again included as a positive control to confirm that P-gp abundance could be regulated in this system. There were no significant changes in hCMEC/D3 abundance of P-gp between any of the ACM treated groups compared to the vehicle control, nor were differences detected between the various ACM treatment groups either (Fig. 7a). Secondly, apoE content within ACM was also analysed via ELISA analysis and this used to approximate a normalised concentration of apoE over the three different isoforms within treatments. A concentration of approximately 162 ng/mL (SD = 7.23 ng/mL) was used based on the apoE content and available volumes after concentrating. After a 72 hour treatment with concentration-corrected ACM, P-gp abundance was unchanged (Fig. 7b). SR12813 treatment did significantly increase P-gp abundance within this setup (Fig. 7c), suggesting that whilst P-gp abundance can be modulated to some extent, treatment with apoE from ACM does not elicit any changes to P-gp abundance.

## Discussion

The BBB is critical in protecting the CNS and in particular, P-gp is a key efflux transporter that regulates the CNS access of drugs. Changes to P-gp abundance and function noted to occur in AD may therefore impact the access of drugs into the CNS. As such, it is essential to identify factors which may influence P-gp function to understand how access of drugs into the CNS may be altered in those with AD. If apoE isoforms were to be involved in changes to P-gp, as has been suggested [35], a detailed understanding of the specific changes occurring would be crucial in targeting affected groups and optimising medication regimens.

Our *in vitro* results to date would indicate that different human apoE isoforms do not directly alter either P-gp abundance or function in the study designs specified. We employed the use of recombinant apoE isoforms to determine the potential direct impact of the protein on P-gp abundance and function *in vitro*. No differences were found between apoE3 or apoE4 isoforms, suggesting that there is no direct impact of apoE isoform on P-gp abundance (Fig. 2a-d). However, the use of SR12183 as a positive control for P-gp regulation confirmed that P-gp abundance could be upregulated in hCMEC/D3 cells (Fig. 2e), supporting a true lack of direct effect of apoE isoforms on P-gp abundance. R123 accumulation studies indicated no change in P-gp function (Fig. 3a). R123 accumulation, rather than permeability, was used to measure P-gp function, as hCMEC/D3 cells exhibit a leaky paracellular route and are not suitable for permeability assays [36]. The accumulation of rhodamine fluorophores in hCMEC/D3 cells is a commonly-reported approach to assess P-gp function [37, 42, 45]. These studies could be complemented with imaging, however, this was not pursued given the accumulation studies demonstrated no impact of apoE isoforms on P-gp function, despite a positive effect with the use of PSC 833 (Fig. 3b).

In its cholesterol trafficking role, apoE interacts with various receptors in the low-density lipoprotein receptor (LDLR) family, including low density lipoprotein receptor-related protein 1 (LRP1) and LDLR [46]. LDLR and LRP1 are both expressed in hCMEC/D3 cells [47, 48]. LRP1 has shown to be expressed on both the abluminal and luminal surfaces of brain endothelial cells [49], whilst LDLR has shown to be luminally expressed [50]. However, LRP1 abundance was shown to be reduced in hCMEC/D3 cells when compared to human brain microvessels [51], and the endocytic functionality of LRP1 in hCMEC/D3 cells has also been questioned relative to other transcytosis mechanisms [52]. Altered receptor functionality may limit the ability of apoE to carry out its physiological function, and the distribution of relevant receptors may be impacted by a possible lack of polarisation in the cell model. However, given the presence of these receptors in the *in vitro* model, should there be an effect to observe, we would expect to see this change. The lipidation status of apoE isoforms is also reported to affect the functionality of the protein, including receptor interactions [53], and apoE lipidation status itself has been postulated as a therapeutic target in AD [54]. Given the recombinant apoE treatments used were not lipidated, this may yield different results to the lipidated versions found in biological systems. The reduction in cell viability with recombinant protein treatments as assessed via MTT (Fig. 1) is also considered a possible contribution to the observed results. The difference in viability between rE3 and rE4 treatments at 10 µg/mL (Fig. 1), whilst suggestive of a toxic effect of apoE4 compared to apoE3, was not borne out in later studies, as cell growth and proliferation were considered normal on visual inspection for all subsequent protein abundance studies, and inter-isoform differences on P-gp were not observed.

To account for some of these limitations with the recombinant protein system, and in an attempt to more closely replicate physiological interactions, treatments using apoE secreted from astrocyte cell culture within ACM were implemented. Here we would expect the apoE isoforms to more closely represent their physiological form, including lipidation by astrocytes upon secretion as has been previously characterised [38]. With the initial use of ACM, it was clear that hCMEC/D3 cell growth and viability were impacted detrimentally compared to hCMEC/D3 cells in their normal growth media (Fig. 4). A similar phenomenon has been reported, where the hCMEC/D3 cell line was subjected to DMEM and cells were observed to have a reduced growth rate [55]. This reduction in viability was lessened when the ACM was diluted out in a 1:2 ratio (Fig. 4). Further, the MTT assay results demonstrated that whilst the vehicle control 1:1 treatments trended toward lower viability compared to basal media, this did not reach the significance of the 1:1 groups containing ACM, suggesting that the conditioning of the media by astrocyte cultures is leading to the observed reductions in hCMEC/D3 cell viability. Astrocytes are reported to modulate important BBB functions through the release of growth factors and cytokines [56], and murine astrocytes have been shown to release a wide array of different proteins [57]. Activated astrocytes have also been implicated in neuronal loss in neurodegenerative disease [58]. Whilst our design did not have astrocytes and endothelial cells in direct communication, it appears likely that a combination of astrocyte-secreted factors has led to the observed reduction in hCMEC/D3 cell viability. Furthermore, the failure of SR12813 in the 1:1 media mix to upregulate P-gp (Fig. 5b), as has been shown in hCMEC/D3 basal growth media (Fig. 5a) and the literature [42], was a key finding in demonstrating that this cell system is not equally modifiable and not valid to accurately assess changes to P-gp abundance.

A method of concentrating ACM was devised using molecular weight cut-off filters to retain apoE collected from astrocytes. Critically, SR12813 was able to significantly increase hCMEC/D3 abundance of P-gp (Fig. 7c) within this new system, showing the ability to modify hCMEC/D3 P-gp abundance in the presence of concentrated media. However, there was no difference in the abundance of P-gp following treatment with conditioned media from different astrocyte isoforms, either when attempting to replicate the 1:1 ratio after concentrating ACM (Fig. 7a) or when normalising apoE content across isoforms (Fig. 7b). This would indicate that although this system is better able to observe changes to P-gp abundance, given the successful use of SR12813, it presents its own limitations. Use of the molecular weight cut-off filters assumes negligible loss of apoE during the concentrating process, and whilst the filtrate was assessed for apoE content and was found to be absent of such, this does not account for apoE that may remain on the filter device itself. Astrocyte cultures also yielded a somewhat lower level of apoE than has previously been characterised [38], thus resulting in treatments with a lower apoE concentration than that of the recombinant studies. Additionally, the small volumes obtained post-concentrating, as well as the high variability of the initial apoE content as assessed by ELISA (Fig. 6a & b), makes controlling apoE levels within final treatments highly challenging.

Changes in P-gp abundance associated with differences in apoE isoform have been observed in FAD mouse models [35], so it remains possible that apoE in combination with AD pathology is required to drive the changes to P-gp reported in the literature. Elsewhere it has been reported that apoE4 causes BBB dysfunction independent of AD pathology [30], although whether this includes impacts on BBB transporters remains unknown. Furthermore, it is possible that apoE-mediated effects impact the BBB through pathways involving other cell types such as pericytes, as has been suggested [31]. The *in vitro* and acute phase nature of these studies may therefore limit the translatability of these findings, which may otherwise be observed in an intact biological system with chronic exposure to the respective apoE isoform, as is the case in humans with AD. In these studies, we used the hCMEC/D3 cell line, an *in vitro* model, which does not account for the potential effects of other components of the neurovascular complex. As such, where feasible, more complex models should be considered by those investigating direct apoE isoform-mediated effects, as there may be indirect effects of apoE on the brain microvasculature initiated through effects on associated neurovascular cells, such as astrocytes and pericytes.

## Conclusion

Under our implemented experiment conditions, there is no direct apoE isoform-dependent influence on either hCMEC/D3 abundance or function of P-gp. These results may have been influenced by model design and be unable to truly encapsulate the chronic nature of AD. However, our results demonstrate a clear lack of effect in this cell system, assisting others in the field seeking to investigate apoE-mediated BBB alterations. Dedicated animal models may be a solution to better assess the direct influence of apoE isoforms on the BBB, particularly with respect to BBB transporters and subsequent effects on drug access into the CNS.

## Data Availability

The data that support the findings of this study are available from the corresponding author upon reasonable request.

## ABBREVIATIONS

ACM: Astrocyte-conditioned media
apoE: Apolipoprotein E
BBB: Blood-brain barrier
*E3*: *APOE3* allele
*E4*: *APOE4* allele
FAD: Familial Alzheimer’s disease
hCMEC/D3: Human cerebral microvascular endothelial cells
P-gp: P-glycoprotein
rE3: Recombinant apolipoprotein E3
rE4: Recombinant apolipoprotein E4
SAD: Sporadic Alzheimer’s disease

## References

[1] Mendez MF. Early-onset Alzheimer disease and its variants. Continuum (Minneap Minn). 2019;25:34–51. 30707186 10.1212/CON.0000000000000687 PMC6538053

[2] Tanzi RE. The genetics of Alzheimer disease. Cold Spring Harb Perspect Med. 2012;2:a006296. 10.1101/cshperspect.a006296 PMC3475404 23028126

[3] Clague F, Mercer SW, McLean G, Reynish E, Guthrie B. Comorbidity and polypharmacy in people with dementia: Insights from a large, population-based cross-sectional analysis of primary care data. Age Ageing. 2017;46:33–9. 28181629 10.1093/ageing/afw176

[4] Chaves JCS, Dando SJ, White AR, Oikari LE. Blood-brain barrier transporters: An overview of function, dysfunction in Alzheimer’s disease and strategies for treatment. Biochim Biophys Acta Mol Basis Dis. 2024;1870:166967. 10.1016/j.bbadis.2023.166967 38008230

[5] Abbott NJ, Patabendige AAK, Dolman DEM, Yusof SR, Begley DJ. Structure and function of the blood–brain barrier. Neurobiol Dis. 2010;37:13–25. 19664713 10.1016/j.nbd.2009.07.030

[6] Begley DJ. ABC transporters and the blood-brain barrier. Curr Pharm Des. 2004;10:1295–312. 15134482 10.2174/1381612043384844

[7] Wang W, Bodles-Brakhop AM, Barger SW. A role for P-glycoprotein in clearance of Alzheimer amyloid β -peptide from the brain. Curr Alzheimer Res. 2016;13:615–20. 26971931 10.2174/1567205013666160314151012 PMC5102249

[8] Mehta DC, Short JL, Nicolazzo JA. Altered brain uptake of therapeutics in a triple transgenic mouse model of Alzheimer’s disease. Pharm Res. 2013;30:2868–79. 23794039 10.1007/s11095-013-1116-2

[9] Hartz AM, Miller DS, Bauer B. Restoring blood-brain barrier P-glycoprotein reduces brain amyloid-beta in a mouse model of Alzheimer’s disease. Mol Pharmacol. 2010;77:715–23. 20101004 10.1124/mol.109.061754 PMC2872973

[10] Park R, Kook SY, Park JC, Mook-Jung I. Aβ1-42 reduces P-glycoprotein in the blood-brain barrier through RAGE-NF-κB signaling. Cell Death Dis. 2014;5:e1299. 10.1038/cddis.2014.258 PMC4611731 24967961

[11] Wijesuriya HC, Bullock JY, Faull RL, Hladky SB, Barrand MA. ABC efflux transporters in brain vasculature of Alzheimer’s subjects. Brain Res. 2010;1358:228–38. 20727860 10.1016/j.brainres.2010.08.034

[12] Jeynes B, Provias J. An investigation into the role of P-glycoprotein in Alzheimer’s disease lesion pathogenesis. Neurosci Lett. 2011;487:389–93. 21047545 10.1016/j.neulet.2010.10.063

[13] Deo AK, Borson S, Link JM, Domino K, Eary JF, Ke B, *et al*. Activity of P-glycoprotein, a β-amyloid transporter at the blood-brain barrier, is compromised in patients with mild Alzheimer disease. J Nucl Med. 2014;55:1106–11. 24842892 10.2967/jnumed.113.130161 PMC4691246

[14] van Assema DME, Lubberink M, Rizzu P, van Swieten JC, Schuit RC, Eriksson J, *et al*. Blood–brain barrier P-glycoprotein function in healthy subjects and Alzheimer’s disease patients: Effect of polymorphisms in the ABCB1 gene. EJNMMI Res. 2012;2:57. 23067778 10.1186/2191-219X-2-57 PMC3483228

[15] Vogelgesang S, Cascorbi I, Schroeder E, Pahnke J, Kroemer HK, Siegmund W, *et al*. Deposition of Alzheimer’s beta-amyloid is inversely correlated with P-glycoprotein expression in the brains of elderly non-demented humans. Pharmacogenetics. 2002;12:535–41. 12360104 10.1097/00008571-200210000-00005

[16] Cirrito JR, Deane R, Fagan AM, Spinner ML, Parsadanian M, Finn MB, *et al*. P-glycoprotein deficiency at the blood-brain barrier increases amyloid-beta deposition in an Alzheimer disease mouse model. J Clin Invest. 2005;115:3285–90. 16239972 10.1172/JCI25247 PMC1257538

[17] Mahley RW, Rall SC Jr. Apolipoprotein E: Far more than a lipid transport protein. Annu Rev Genomics Hum Genet. 2000;1:507–37. 11701639 10.1146/annurev.genom.1.1.507

[18] Farrer LA, Cupples LA, Haines JL, Hyman B, Kukull WA, Mayeux R, *et al*. Effects of age, sex, and ethnicity on the association between apolipoprotein E genotype and Alzheimer disease. A meta-analysis. ApoE and Alzheimer disease meta analysis consortium. JAMA. 1997;278:1349–56. 9343467 10.1001/jama.1997.03550160069041

[19] Elshourbagy NA, Liao WS, Mahley RW, Taylor JM. Apolipoprotein E mRNA is abundant in the brain and adrenals, as well as in the liver, and is present in other peripheral tissues of rats and marmosets. Proc Natl Acad Sci USA. 1985;82:203–7. 3918303 10.1073/pnas.82.1.203 PMC397000

[20] Xu Q, Bernardo A, Walker D, Kanegawa T, Mahley RW, Huang Y. Profile and regulation of apolipoprotein E (ApoE) expression in the CNS in mice with targeting of green fluorescent protein gene to the ApoE locus. J Neurosci. 2006;26:4985–94. 16687490 10.1523/JNEUROSCI.5476-05.2006 PMC6674234

[21] Linton MF, Gish R, Hubl ST, Bütler E, Esquivel C, Bry WI, *et al*. Phenotypes of apolipoprotein B and apolipoprotein E after liver transplantation. J Clin Invest. 1991;88:270–81. 2056122 10.1172/JCI115288 PMC296029

[22] Liu M, Kuhel DG, Shen L, Hui DY, Woods SC. Apolipoprotein E does not cross the blood-cerebrospinal fluid barrier, as revealed by an improved technique for sampling CSF from mice. Am J Physiol Regul Integr Comp Physiol. 2012;303:R903-8. 22933021 10.1152/ajpregu.00219.2012 PMC3517701

[23] Holtzman DM, Bales KR, Tenkova T, Fagan AM, Parsadanian M, Sartorius LJ, *et al*. Apolipoprotein E isoform-dependent amyloid deposition and neuritic degeneration in a mouse model of Alzheimer’s disease. Proc Natl Acad Sci USA. 2000;97:2892–7. 10694577 10.1073/pnas.050004797 PMC16026

[24] Castellano JM, Kim J, Stewart FR, Jiang H, DeMattos RB, Patterson BW, *et al*. Human apoE isoforms differentially regulate brain amyloid-β peptide clearance. Sci Transl Med. 2011;3:89ra57. 10.1126/scitranslmed.3002156 PMC3192364 21715678

[25] Deane R, Sagare A, Hamm K, Parisi M, Lane S, Finn MB, *et al*. ApoE isoform-specific disruption of amyloid beta peptide clearance from mouse brain. J Clin Invest. 2008;118:4002–13. 19033669 10.1172/JCI36663 PMC2582453

[26] Ossenkoppele R, Jansen WJ, Rabinovici GD, Knol DL, van der Flier WM, van Berckel BN, *et al*. Prevalence of amyloid PET positivity in dementia syndromes: A meta-analysis. JAMA. 2015;313:1939–49. 25988463 10.1001/jama.2015.4669 PMC4517678

[27] Shi Y, Yamada K, Liddelow SA, Smith ST, Zhao L, Luo W, *et al*. ApoE4 markedly exacerbates tau-mediated neurodegeneration in a mouse model of tauopathy. Nature. 2017;549:523–7. 28959956 10.1038/nature24016 PMC5641217

[28] Bell RD, Winkler EA, Singh I, Sagare AP, Deane R, Wu Z, *et al*. Apolipoprotein E controls cerebrovascular integrity via cyclophilin A. Nature. 2012;485:512–6. 22622580 10.1038/nature11087 PMC4047116

[29] Liu CC, Yamazaki Y, Heckman MG, Martens YA, Jia L, Yamazaki A, *et al*. Tau and apolipoprotein E modulate cerebrovascular tight junction integrity independent of cerebral amyloid angiopathy in Alzheimer’s disease. Alzheimers Dement. 2020;16:1372–83. 32827351 10.1002/alz.12104 PMC8103951

[30] Montagne A, Nation DA, Sagare AP, Barisano G, Sweeney MD, Chakhoyan A, *et al*. APOE4 leads to blood-brain barrier dysfunction predicting cognitive decline. Nature. 2020;581:71–6. 32376954 10.1038/s41586-020-2247-3 PMC7250000

[31] Montagne A, Nikolakopoulou AM, Huuskonen MT, Sagare AP, Lawson EJ, Lazic D, *et al*. APOE4 accelerates advanced-stage vascular and neurodegenerative disorder in old Alzheimer’s mice via cyclophilin A independently of amyloid-β. Nat Aging. 2021;1:506–20. 35291561 10.1038/s43587-021-00073-z PMC8920485

[32] Halliday MR, Rege SV, Ma Q, Zhao Z, Miller CA, Winkler EA, *et al*. Accelerated pericyte degeneration and blood-brain barrier breakdown in apolipoprotein E4 carriers with Alzheimer’s disease. J Cereb Blood Flow Metab. 2016;36:216–27. 25757756 10.1038/jcbfm.2015.44 PMC4758554

[33] Barisano G, Kisler K, Wilkinson B, Nikolakopoulou AM, Sagare AP, Wang Y, *et al*. A “multi-omics” analysis of blood-brain barrier and synaptic dysfunction in APOE4 mice. J Exp Med. 2022;219:e20221137. 10.1084/jem.20221137 PMC9435921 36040482

[34] Alata W, Ye Y, St-Amour I, Vandal M, Calon F. Human apolipoprotein E ɛ4 expression impairs cerebral vascularization and blood-brain barrier function in mice. J Cereb Blood Flow Metab. 2015;35:86–94. 25335802 10.1038/jcbfm.2014.172 PMC4296574

[35] Lin AL, Parikh I, Yanckello LM, White RS, Hartz AMS, Taylor CE, *et al*. APOE genotype-dependent pharmacogenetic responses to rapamycin for preventing Alzheimer’s disease. Neurobiol Dis. 2020;139:104834. 32173556 10.1016/j.nbd.2020.104834 PMC7486698

[36] Weksler B, Romero IA, Couraud PO. The hCMEC/D3 cell line as a model of the human blood brain barrier. Fluids Barriers CNS. 2013;10:16. 23531482 10.1186/2045-8118-10-16 PMC3623852

[37] Pyun J, McInnes LE, Donnelly PS, Mawal C, Bush AI, Short JL, *et al*. Copper bis(thiosemicarbazone) complexes modulate P-glycoprotein expression and function in human brain microvascular endothelial cells. J Neurochem. 2022;162:226–44. 35304760 10.1111/jnc.15609 PMC9540023

[38] Morikawa M, Fryer JD, Sullivan PM, Christopher EA, Wahrle SE, DeMattos RB, *et al*. Production and characterization of astrocyte-derived human apolipoprotein E isoforms from immortalized astrocytes and their interactions with amyloid-beta. Neurobiol Dis. 2005;19:66–76. 15837562 10.1016/j.nbd.2004.11.005

[39] Riddell DR, Zhou H, Atchison K, Warwick HK, Atkinson PJ, Jefferson J, *et al*. Impact of apolipoprotein E (ApoE) polymorphism on brain ApoE levels. J Neurosci. 2008;28:11445–53. 18987181 10.1523/JNEUROSCI.1972-08.2008 PMC6671315

[40] Wahrle SE, Shah AR, Fagan AM, Smemo S, Kauwe JS, Grupe A, *et al*. Apolipoprotein E levels in cerebrospinal fluid and the effects of ABCA1 polymorphisms. Mol Neurodegener. 2007;2:7. 17430597 10.1186/1750-1326-2-7 PMC1857699

[41] Cruchaga C, Kauwe JS, Nowotny P, Bales K, Pickering EH, Mayo K, *et al*. Cerebrospinal fluid APOE levels: An endophenotype for genetic studies for Alzheimer’s disease. Hum Mol Genet. 2012;21:4558–71. 22821396 10.1093/hmg/dds296 PMC3459471

[42] Zastre JA, Chan GN, Ronaldson PT, Ramaswamy M, Couraud PO, Romero IA, *et al*. Up-regulation of P-glycoprotein by HIV protease inhibitors in a human brain microvessel endothelial cell line. J Neurosci Res. 2009;87:1023–36. 18855943 10.1002/jnr.21898

[43] Chan GN, Patel R, Cummins CL, Bendayan R. Induction of P-glycoprotein by antiretroviral drugs in human brain microvessel endothelial cells. Antimicrob Agents Chemother. 2013;57:4481–8. 23836171 10.1128/AAC.00486-13 PMC3754350

[44] Atadja P, Watanabe T, Xu H, Cohen D. PSC-833, a frontier in modulation of P-glycoprotein mediated multidrug resistance. Cancer Metastasis Rev. 1998;17:163–8. 9770112 10.1023/A:1006046201497

[45] Alms D, Fedrowitz M, Römermann K, Noack A, Löscher W. Marked differences in the effect of antiepileptic and cytostatic drugs on the functionality of P-glycoprotein in human and rat brain capillary endothelial cell lines. Pharm Res. 2014;31:1588–604. 24477677 10.1007/s11095-013-1264-4

[46] Kanekiyo T, Xu H, Bu G. ApoE and Aβ in Alzheimer’s disease: Accidental encounters or partners? Neuron. 2014;81:740–54. 24559670 10.1016/j.neuron.2014.01.045 PMC3983361

[47] Kakava S, Schlumpf E, Panteloglou G, Tellenbach F, von Eckardstein A, Robert J. Brain endothelial cells in contrary to the aortic do not transport but degrade low-density lipoproteins via both LDLR and ALK1. Cells. 2022;11:3044. 10.3390/cells11193044 PMC9564369 36231005

[48] András IE, Eum SY, Huang W, Zhong Y, Hennig B, Toborek M. HIV-1-induced amyloid beta accumulation in brain endothelial cells is attenuated by simvastatin. Mol Cell Neurosci. 2010;43:232–43. 19944163 10.1016/j.mcn.2009.11.004 PMC2818553

[49] Tian X, Leite DM, Scarpa E, Nyberg S, Fullstone G, Forth J, *et al*. On the shuttling across the blood-brain barrier via tubule formation: Mechanism and cargo avidity bias. Sci Adv. 2020;6:eabc4397. 10.1126/sciadv.abc4397 PMC7695481 33246953

[50] Molino Y, David M, Varini K, Jabès F, Gaudin N, Fortoul A, *et al*. Use of LDL receptor—targeting peptide vectors for in vitro and in vivo cargo transport across the blood-brain barrier. FASEB J. 2017;31:1807–27. 28108572 10.1096/fj.201600827R

[51] Ohtsuki S, Ikeda C, Uchida Y, Sakamoto Y, Miller F, Glacial F, *et al*. Quantitative targeted absolute proteomic analysis of transporters, receptors and junction proteins for validation of human cerebral microvascular endothelial cell line hCMEC/D3 as a human blood-brain barrier model. Mol Pharm. 2013;10:289–96. 23137377 10.1021/mp3004308

[52] Ito S, Oishi M, Ogata S, Uemura T, Couraud PO, Masuda T, *et al*. Identification of cell-surface proteins endocytosed by human brain microvascular endothelial cells in vitro. Pharmaceutics. 2020;12:579. 10.3390/pharmaceutics12060579 PMC7356521 32585920

[53] Bu G. Apolipoprotein E and its receptors in Alzheimer’s disease: Pathways, pathogenesis and therapy. Nat Rev Neurosci. 2009;10:333–44. 19339974 10.1038/nrn2620 PMC2908393

[54] Lanfranco MF, Ng CA, Rebeck GW. ApoE lipidation as a therapeutic target in Alzheimer’s disease. Int J Mol Sci. 2020;21:6336. 10.3390/ijms21176336 PMC7503657 32882843

[55] Hinkel S, Mattern K, Dietzel A, Reichl S, Müller-Goymann CC. Parametric investigation of static and dynamic cell culture conditions and their impact on hCMEC/D3 barrier properties. Int J Pharm. 2019;566:434–44. 31163193 10.1016/j.ijpharm.2019.05.074

[56] Abbott NJ, Rönnbäck L, Hansson E. Astrocyte-endothelial interactions at the blood-brain barrier. Nat Rev Neurosci. 2006;7:41–53. 16371949 10.1038/nrn1824

[57] Dowell JA, Johnson JA, Li L. Identification of astrocyte secreted proteins with a combination of shotgun proteomics and bioinformatics. J Proteome Res. 2009;8:4135–43. 19469553 10.1021/pr900248y PMC2866504

[58] Liddelow SA, Guttenplan KA, Clarke LE, Bennett FC, Bohlen CJ, Schirmer L, *et al*. Neurotoxic reactive astrocytes are induced by activated microglia. Nature. 2017;541:481–7. 28099414 10.1038/nature21029 PMC5404890

